# Realizing the value of complex medical technology: Demonstrating cost-effectiveness is not enough

**DOI:** 10.1016/j.ctro.2023.100644

**Published:** 2023-06-01

**Authors:** C. Hehakaya, G.W.J. Frederix, J.RN. van der Voort van Zyp, D.E. Grobbee, H.M. Verkooijen

**Affiliations:** a Global Public Health & Bioethics, Julius Center for Health Sciences and Primary Care, University Medical Center Utrecht, Utrecht, The Netherlands; b Health Economic Evaluation, Julius Center for Health Sciences and Primary Care, University Medical Center Utrecht, Utrecht, The Netherlands; c Division of Imaging & Oncology, University Medical Center Utrecht, Utrecht, The Netherlands; d Utrecht University, The Netherlands

**Keywords:** Technologies, Innovations, Implementation, Stakeholders, Complex interventions, Evaluation

## Abstract

The main factors driving the value of medical treatments are proven effectiveness and cost-effectiveness. This is different for complex medical technologies that combine scientific disciplines, functions or tools in a single solution-oriented method. This short communication provides three recommendations to realize the value of complex medical technologies. It is important to engage stakeholders before technology implementation to promote its relevance from multiple perspectives, to explore opportunities for professional development and collaboration, and to demonstrate the societal effects over the entire life-cycle.

## Highlights

• Engaging stakeholders before implementing the complex medical technology is important to promote its relevance, usefulness and effectiveness from multiple perspectives.
• Exploring the potential of new complex medical technology for professional development and collaboration is essential to get a better picture of its potential value.
• Demonstrating the individual and societal effects of the technology over the entire life-cycle will help to understand its long-term impact.

Traditionally, the main factors driving the value of medical treatments are proven effectiveness and cost-effectiveness [1]. This is different for complex medical technologies that combine scientific disciplines, functions or tools in a single solution-oriented method. These technologies frequently combine clinical, technical, economic, organizational and social synergies for disease prevention, diagnosis, monitoring or therapy [2]. Patients should be at the center when introducing complex medical technologies, yet, implementation also affects healthcare professionals, providers, researchers, payers, the manufacturing industry, and governmental bodies [3], [4].

The MR-Linac illustrates a complex technology by integrating two traditionally separate modalities, diagnostic MRI guidance and radiation therapy into one device for cancer treatment [5]. This, together with digitisation in the MR-Linac technology, requires different knowledge bases and competences of involved actors. Implementing complex medical technologies needs to be more than a technical and clinical exercise to achieve their potential. Based on research articles on MR-Linac technology, three recommendations can be made:

## 1 Engage and empower stakeholders before implementation

First, it is important to engage stakeholders before implementing the complex medical technology in clinical practice to promote its relevance, usefulness and effectiveness from multiple perspectives. For instance, employing complex medical technologies requires collaboration between individuals with diverse expertise and backgrounds such as healthcare professionals from different medical specialties, physicists, ICT experts and referring physicians [3], [6]. This may change traditional clinical practice and the working tasks of those involved. It is therefore important to acknowledge that each individual may have different perspectives on characterizing the impact of implementation. This provides opportunities to bridge differences and increase mutual understanding. Early dialogue between individuals is therefore necessary to promote implementation and can tackle organizational barriers such as professional silos, resistance to changing working tasks, ethical deliberations, conflicts of interests, inefficiencies and erosion of trust among physicians. A shift from the traditional medical practice in a soloed culture towards a more open, collaborative culture is crucial. Therefore, healthcare professionals need to embrace a team-based attitude in which values and principles are shared and communicated among team members.

Second, it is important to engage stakeholders before implementation to encourage them to take timely and informed implementation steps. Complex medical technologies will transform conventional clinical practice and impact a hospital’s administrative processes [3], [4]. Early dialogues between healthcare professionals and external institutions (e.g., payers, manufacturers, governance bodies) is therefore necessary to assure institutional-level knowledge and for processing technological, economic and regulatory changes in time. These could include approved authorizations for new advanced techniques and appropriate policies for new roles and responsibilities of staff. For instance, early engagement with national and international professional associations is necessary to formulate and update recommendations of clinical practice guidelines as they are major drivers of clinical practice. These associations also play an important role in encouraging healthcare providers to put words into actions. Moreover, engaging health insurers and governing bodies may help to establish the right incentives for encouraging healthcare professionals working together and demonstrating the impact of technology.

During stakeholder engagement, interaction between individuals is crucial to understand their different interests and needs, support and attitude, and influence on technology implementation. These insights need to guide implementation and define metrics to make data-supported decisions. Co-design and participatory approaches (e.g., semi-structured interviews, workshops) can facilitate engagement and provide the necessary formation for further technology design and implementation.

## 2 Seize the opportunity of professional development and best practices

It is important to explore opportunities for professional development and collaboration. Complex medical technologies may be attractive to healthcare workers such as physicians, technicians and nurses to acquire new knowledge and skills, obtain increased autonomy, and further develop tasks and responsibilities [3], [4]. Moreover, early technology adopters are in a unique position to help later adopters and ease implementation challenges of complex medical technologies, such as the required technical expertise, staff investments, necessary infrastructure changes and financial trade-offs. The early adopter experience provides valuable insights into best practices and may inform the design and dissemination program for future technology implementation. This is especially useful for advanced technologies that are still developing, and thus with changing patterns of use. Best practices would show the best way to deploy the complex medical technology effectively, and have been worked out through trial and error.

Complex medical technologies with digitization may allow remote treatment, treatment planning and supervision. Early adopters could assist later adopters in clinical decision-making, education or training simulations remotely. A digital clinical infrastructure, developed at an accelerated pace during the COVID-19 pandemic [7], can centralize knowledge and clinical decision-making, and enable a virtual community in which physicians execute decisions quickly and in a unified manner. This chain of command reduces the duplication of responsibilities that may result in additional costs to the later adopter, reducing implementation waste and burden. Moreover, standardized procedures and better supervision in centralized clinical decision-making led by early adopters, can also result in improved quality of work [8]. This would reduce traveling making it more convenient for providers, and improves healthcare access for patients in rural and remote areas.

An interdisciplinary consortium is important to explore clinical, technical, moral, economic, social, political and environmental aspects of implementation in an integrative way. Consortia may act as an information and communication ecosystem [5], which ideally supports technology development and assessment, early effectiveness studies, (early) health economic analyses, randomized controlled trials, knowledge dissemination, and the exchange of best practices at both the individual and institutional level. This consortium can unify early and later technology adopters, and facilitate dialogues between relevant stakeholders.

## 3 Demonstrate individual and societal benefits continuously

To effectively use the potential of complex medical technology for relevant stakeholders, it is important to demonstrate the societal effects that implementation has on its environment over the entire period of its life-cycle. Complex medical technologies can transform independent healthcare activities into a single activity organization to generate economies of scale [9]. It is therefore essential to identify which working tasks and clinical practices will be replaced and reduced as a result of implementation. Moreover, it is important to demonstrate and exploit the opportunities of professional development which can increase work satisfaction and a sense of belonging.

Health economic evaluation and modelling early in the development and throughout the evaluation process would be useful, including hybrid designs that seek to jointly test impact on implementation and societal effects. Health economic modelling is a relatively low-cost and quick method to not only estimate cost-effectiveness in later stages, but also examine the potential impact of an innovation. This approach is especially useful for advanced technologies with digital development, where the value proposition is not straightforward and evolves along with digitization [10]. A detailed consideration is necessary including all resource implications, ‘hidden’ costs related to all implementation activities (e.g., manualizing the technology, costs of developing and delivering education, and train-the-trainer interventions) and broader societal effects. Insights gathered through stakeholder engagement, as recommended earlier, can pave the way for discussing relevant societal effects.

## 4 Conclusions

As clear as the need may be for demonstrating the societal effects of complex medical technology, a big challenge is to start assessing these effects, and to realize potential benefits. Working in healthcare is, at its core, caring for patients. Demonstrating societal effects is thus often experienced as “extra work” with additional administrative tasks. However, complex medical technologies have the potential to improve healthcare workers’ workload and emotional distress which have been substantially increased since the start of the COVID-19 pandemic. Clinical research on effectiveness and cost-effectiveness in implementation is still important, but not enough to realize the value of complex medical technologies: early stakeholder engagement, professional development and new modes of collaboration are just as relevant.

## Funding

This project is being funded by the Netherlands Organization for Health Research and Development (ZonMw) via Innovative Medical Devices Initiative for Technology for Sustainable Healthcare under the project title ‘Clinical introduction online and real-time MRI-guided prostate cancer radiotherapy’ (project number 104006004).

## Declaration of Competing Interest

The authors declare that they have no known competing financial interests or personal relationships that could have appeared to influence the work reported in this paper.

## References

[1] Van Loon J., Grutters J., Macbeth F. (2012). Evaluation of novel radiotherapy technologies: What evidence is needed to assess their clinical and cost effectiveness, and how should we get it?. Lancet Oncol.

[2] Phillips M., Harrington T., Srai J. (2017). Convergent innovation in emerging healthcare technology ecosystems: addressing complexity and integration. Technol Innov Manag Rev.

[3] Hehakaya C., Van der Voort van Zyp J.R., Lagendijk J.J.W., Grobbee D.E., Verkooijen H.M., Moors E.H.M. (2020). Problems and promises of introducing the magnetic resonance imaging linear accelerator into routine care: the case of prostate cancer. Front Oncol.

[4] Hehakaya C., Sharma A.M., van der Voort Van Zijp J.R.N., Grobbee D.E., Verkooijen H.M., Izaguirre E.W. (2022). Implementation of magnetic resonance imaging-guided radiation therapy in routine care: opportunities and challenges in the United States. Adv Radiat Oncol.

[5] Kerkmeijer L.G.W., Fuller C.D., Verkooijen H.M., Verheij M., Choudhury A., Harrington K.J. (2016). The MRI-linear accelerator consortium: Evidence-based clinical introduction of an innovation in radiation oncology connecting researchers, methodology, data collection, quality assurance, and technical development. Front Oncol.

[6] Jha S.K., Mcdermott J., Bacon G., Lannon C., Joshi P.K., Dubé L. (2014). Convergent innovation for affordable nutrition, health, and health care: the global pulse roadmap. Ann N Y Acad Sci.

[7] Shu D., Ting W., Carin L., Dzau V., Wong T.Y. (2020). Digital technology and COVID-19. Nat Med.

[8] Cartier B.Y., Fichtenberg C., Gottlieb L.M. (2020). Implementing community resource referral technology: facilitators and barriers described by early adopters. Health Aff.

[9] Dubé L. (2014). Convergent innovation for sustainable economic growth and affordable universal health care: innovating the way we innovate. Ann N Y Acad Sci.

[10] Hehakaya C. (2021). Early health economic analysis of 1.5T MRI-guided radiotherapy for localized prostate cancer: decision analytic modelling. Radiother Oncol.

